# Perianal Endometriosis: An Uncommon Site for a Common Problem

**DOI:** 10.7759/cureus.44840

**Published:** 2023-09-07

**Authors:** Katie McComb, Mohammed Barghash, Saleh Eltayef

**Affiliations:** 1 General Surgery, North Manchester General Hospital, Manchester, GBR; 2 Surgery, North Manchester General Hospital, Manchester, GBR; 3 General and Colorectal Surgery, North Manchester General Hospital, Manchester, GBR

**Keywords:** pain, pelvic, perianal, episiotomy, endometriosis

## Abstract

Endometriosis is the presence of uterine glands and stroma outside of the uterus. It is highly prevalent in women of reproductive age. It is usually found in the pelvis, with most cases being found on the peritoneum, ovaries, or deep in the pelvis. Extraperitoneal endometriosis is uncommon. Perianal endometriosis has an incidence of only 0.2%. We present the case of a 37-year-old woman with recurrent pain and swelling in the perineum at the site of a previous episiotomy scar. Initial imaging and assessment determined this to be scar tissue. Following re-presentation, it was mistakenly diagnosed as a perianal abscess, and the patient underwent incision and drainage. The wound failed to heal with significant induration. Further assessment of the wound was undertaken under general anesthesia. An excision of the affected area was performed, with histological analysis confirming endometriosis. This case highlights that extra-peritoneal endometriosis is a rare but treatable cause of recurrent, cyclical pelvic pain and swelling in the perineum. A high index of clinical suspicion is required due to its ability to mimic other pathologies, including abscesses and cysts. The primary management of perianal endometriosis is surgical excision. Where complete excision is not possible, medical management with hormone therapy should be considered.

## Introduction

Endometriosis is the presence of endometrial glands and stromal tissue outside the uterus [1]. It commonly presents in women of reproductive age, with a prevalence of 190 million women globally [2]. It is the second most common gynecological condition in the United Kingdom (UK) [3].

It is an estrogen-dependent inflammatory disease [4]. Symptoms commonly include cyclical and noncyclical pelvic pain, severe menstrual pain, dyspareunia, heavy menstruation, fatigue, depression, altered bowel habits, and infertility [2,5]. These symptoms overlap with many other conditions, which often leads to a delay in diagnosis [2,5].

The perineum refers to the area between the posterior commissure of the labia majora and the anus in women. Perineal masses can include benign pathologies such as perineal cysts, abscesses, and hemorrhoids, as well as malignant soft tissue masses [6]. In this article, we describe a case of perianal endometriosis presenting as a recurrent painful mass in the perineum.

## Case presentation

A 37-year-old woman presented to a gynecology clinic with a one-year history of a perineal lump. She reported pain in movement and exacerbations of symptoms during menstruation. Her obstetric history consisted of six pregnancies with spontaneous vaginal delivery and one previous episiotomy. She denied any associated vaginal discharge, dyspareunia, urinary, or bowel symptoms.

**Figure 1:**
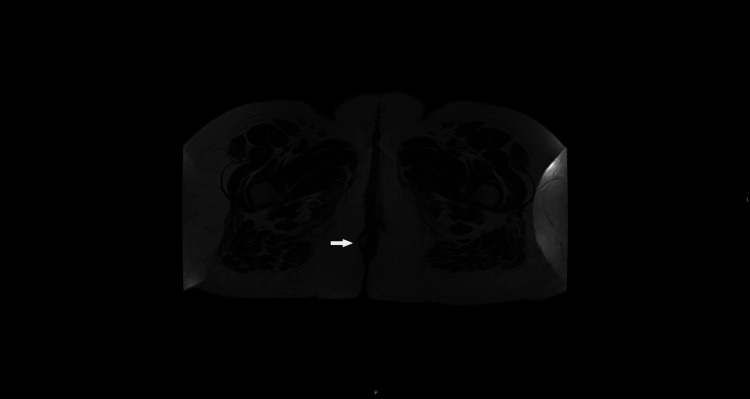
T2-weighted MRI scan of the pelvis axial section with an arrow demonstrating a small lesion in the perianal area

**Figure 2:**
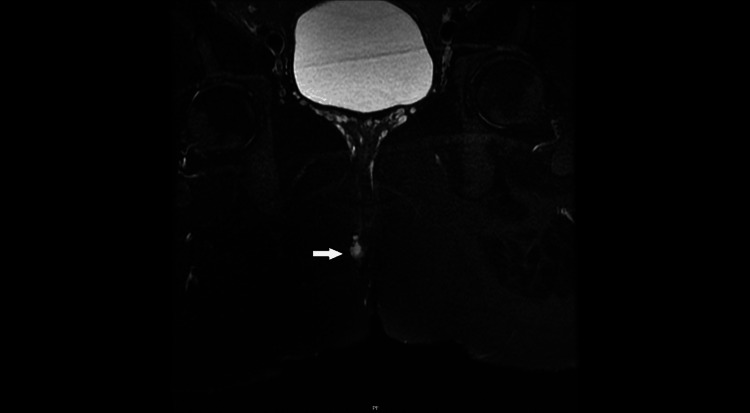
T2 fat-saturated MRI scan of the pelvis coronal section with an arrow demonstrating a heterogenous signal indicating a small perianal lesion

The examination of her abdomen and rectum was unremarkable. There was no evidence of fistulae, fissures, or hemorrhoids. A 1-cm tender swelling was noted directly beneath a previous episiotomy scar. Magnetic resonance imaging (MRI) was arranged and revealed a non-specific lesion in the perineum (Figures 1, 2). The gynecology team referred the patient to the colorectal team. Following their review, a diagnosis of scar tissue was made, and the patient was discharged.

**Figure 3:**
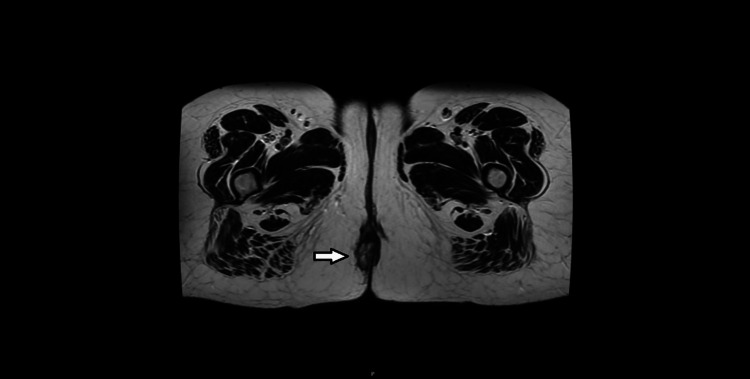
Axial T2-weighted MRI scan of the pelvis with an arrow demonstrating a heterogeneous lobulated lesion in the perianal area

**Figure 4:**
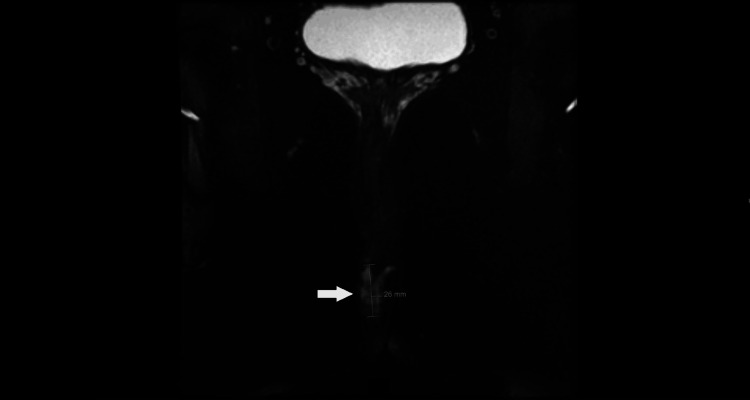
Coronal T2 fat-saturated MRI image displaying a high T2 signal side branch extending across the midline anteriorly and ending blindly on the left side

The patient re-presented to the colorectal surgery clinic five years later. She reiterated the same symptoms of tender perineal swelling aggravated by menstruation. She denied any other symptoms. A further MRI scan was arranged, which demonstrated a heterogeneous T2 signal in the perianal area, measuring 2.4cm x 1.1cm x 2.5cm. Additionally, a high T2 signal side branch was seen extending across the midline anteriorly and ending blindly on the left side. There was a low T2 signal around the heterogeneous signal, which suggested fibrosis. Based on these changes, the radiologist reported that this was likely to represent a chronic abscess (Figures 3, 4).

However, due to the cyclical nature of the patient’s symptoms, subcutaneous endometrial deposits remained within the differential diagnosis. Due to this diagnostic uncertainty, the patient was listed for an elective examination under anesthesia (EUA) to enable further assessment and management.

While awaiting elective admission, she presented to the Accident and Emergency Department with a 3 cm x 4 cm tender swelling in the peri-anal region. A provisional diagnosis of a perianal abscess was made by the General Surgery team. She was taken to the theater for EUA, incision, and drainage. There were no internal openings or masses noted at EUA. A perianal incision was made over the area of fluctuance, and a very small amount of infected blood was drained.

Postoperatively, the wound remained open for two months, with evidence of an underlying area of induration. She was relisted for a further EUA and excision of the area of concern. Due to the complexity of the case, both colorectal and gynecology consultants attended. The area was excised with 2-millimeter margins. The medial end of the lesion was closely related to the external anal sphincter; the margin was not completely achieved.

On histological assessment, anal skin with extensive endometriosis in the dermis and subcutis, associated with decidual alteration and acute inflammation, was reported. There was no atypia or malignancy.

At review postoperatively, the wound had healed well. The patient reported only minor bleeding at the wound site during menses but no further pain. Further excision of this area was not recommended due to the high risk of sphincter injury and the almost complete resolution of the patient’s symptoms. Consequently, she was referred to the gynecology team for the implementation of non-surgical management.

## Discussion

Endometriosis is most commonly found in the pelvic region, with 80% of cases being superficial peritoneal endometriosis. Other pelvic subtypes include ovarian endometriosis and deep endometriosis. More rarely, extraperitoneal endometriosis has been documented in the upper abdominal viscera, diaphragm, pleura, abdominal wall, and nervous system [4,5,7]. Perianal endometriosis is extremely rare, with an incidence of 0.2% [8]. On review, 22 case reports have been published documenting perianal endometriosis [8]. These cases have been reported after obstetric or gynecological procedures, usually at the site of episiotomy scars [7,8].

The etiology of endometriosis is uncertain, particularly when it is extraperitoneal. The most commonly accepted theory is that of retrograde menstruation, whereby the endometrial cells enter the pelvic cavity and adhere to peritoneal surfaces [4]. However, this theory alone does not explain the occurrence of extraperitoneal endometriosis. In the case of perianal endometriosis, it is postulated that endometrial cells can implant in the perineum during vaginal delivery [8,9]. This includes the episiotomy site [8-10]. In this case, the location of the endometriosis at the site of the previous episiotomy scar supports this mechanism of pathogenesis.

A definitive diagnosis is usually made through visualization during surgery. Imaging modalities such as ultrasound and MRI have been found to be inaccurate compared to surgical evaluation but can assist operative planning [5,8]. The challenge of diagnosis based on imaging was clearly highlighted in our case. Imaging of the endometrioma was misinterpreted, which reinforces the importance of surgical evaluation and histological analysis of tissue to confirm the diagnosis.

Endometriosis is initially managed with simple analgesia, including paracetamol and non-steroidal anti-inflammatory drugs, and hormonal treatments such as the combined oral contraceptive pill [2]. Further management options can include the use of gonadotrophin-releasing hormone agonists and antagonists, as well as surgical excision or ablation [2]. Perianal endometriosis has been shown to be best managed with a primarily surgical approach. Wide local excision of the endometrioma and sparing the anal sphincter can be curative [7,9,10]. If the endometriosis involves the anal sphincter, this can be resected; however, sphincteroplasty is then required due to the risk of incontinence [11,12]. Hormonal therapy is effective where there are multiple sites or where complete resection cannot be achieved [9].

## Conclusions

This case highlights the importance of considering extraperitoneal endometriosis as a cause of persistent, recurrent, and cyclical pelvic pain and swelling in women of reproductive age. It should be strongly considered if the site of the pain is related to a previous episiotomy scar. A strong clinical suspicion is required due to its ability to mimic other pathologies, such as perianal abscesses. A multidisciplinary approach is needed to facilitate effective medical and surgical management of these cases.
